# Transcription of hepatitis B surface antigen shifts from cccDNA to integrated HBV DNA during treatment

**DOI:** 10.1172/JCI184243

**Published:** 2025-01-30

**Authors:** Maraake Taddese, Tanner Grudda, Giulia Belluccini, Mark Anderson, Gavin Cloherty, Hyon S. Hwang, Monika Mani, Che-Min Lo, Naomi Esrig, Mark S. Sulkowski, Richard K. Sterling, Yang Zhang, Ruy M. Ribeiro, David L. Thomas, Chloe L. Thio, Ashwin Balagopal

**Affiliations:** 1 Department of Medicine, Johns Hopkins University School of Medicine, Baltimore, Maryland, USA.; 2 Department of Molecular Microbiology and Immunology, Johns Hopkins Bloomberg School of Public Health, Johns Hopkins University, Baltimore, Maryland, USA.; 3 Los Alamos National Laboratory, Los Alamos, New Mexico, USA.; 4 Abbott Diagnostics, Abbott Park, Illinois, USA.; 5 Divison of Gastroenterology, Hepatology, and Nutrition, Virginia Commonwealth University, Richmond, Virginia, USA.; 6 Division of Gastrointestinal and Hepatic Pathology, Joint Pathology Center, Silver Spring, Maryland, USA.

**Keywords:** Infectious disease, Virology, Hepatitis, Transcription

## Abstract

The cornerstone of functional cure for chronic hepatitis B (CHB) is hepatitis B surface antigen (HBsAg) loss from blood. HBsAg is encoded by covalently closed circular DNA (cccDNA) and HBV DNA integrated into the host genome (iDNA). Nucleos(t)ide analogs (NUCs), the mainstay of CHB treatment, rarely lead to HBsAg loss, which we hypothesized was due to continued iDNA transcription despite decreased cccDNA transcription. To test this, we applied a multiplex droplet digital PCR that identifies the dominant source of HBsAg mRNAs to 3,436 single cells from paired liver biopsies obtained from 10 people with CHB and HIV receiving NUCs. With increased NUC duration, cells producing HBsAg mRNAs shifted their transcription from chiefly cccDNA to chiefly iDNA. This shift was due to both a reduction in the number of cccDNA-containing cells and diminished cccDNA-derived transcription per cell; furthermore, it correlated with reduced detection of proteins deriving from cccDNA but not iDNA. Despite this shift in the primary source of HBsAg, rare cells remained with detectable cccDNA-derived transcription, suggesting a source for maintaining the replication cycle. Functional cure must address both iDNA and residual cccDNA transcription. Further research is required to understand the significance of HBsAg when chiefly derived from iDNA.

## Abstract 2

Using a novel ddPCR assay in single cells, we show that the main source of hepatitis B surface mRNAs shifts from cccDNA to iDNA with NUC treatment.

## Graphical abstract

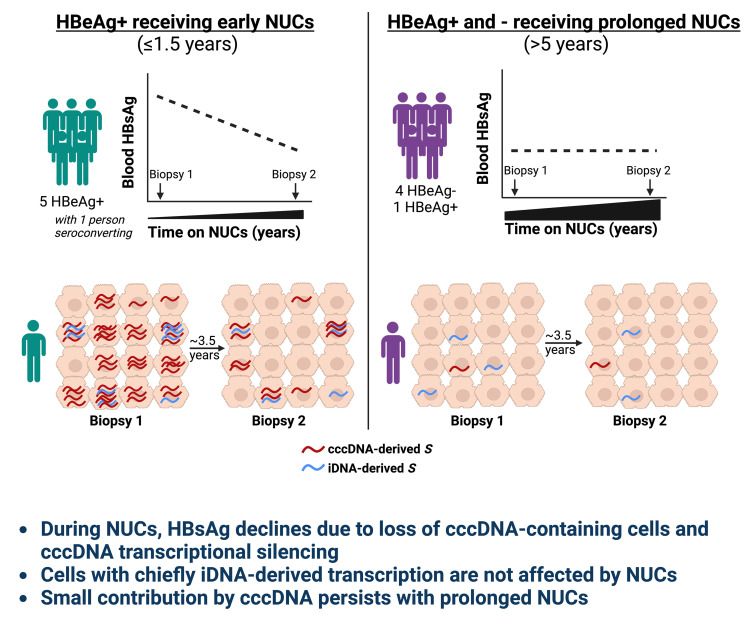

## Introduction

Chronic hepatitis B (CHB), which affects an estimated 300 million people worldwide, is a leading cause of hepatocellular carcinoma and end-stage liver disease. Although CHB can be treated with pegylated interferon-α and/or nucleos(t)ide analogs (NUCs), treatment typically only suppresses production of HBV DNA and rarely leads to loss of hepatitis B surface antigen (HBsAg) from blood, which is essential for meeting the current guidelines of a functional cure (1). Surface (*S*) mRNAs that are translated into HBsAg are transcribed from 2 sources: the covalently closed circular DNA (cccDNA) and HBV DNA that is integrated into the host genome (iDNA). It is important to understand the dominant source of transcription during treatment to uncover how HBsAg persists in most people despite suppression of HBV DNA production. Moreover, the dynamics of the HBsAg source during treatment are key for targeting novel therapies and understanding deficiencies of existing therapies. Such data are limited because existing clinical blood markers do not distinguish between these sources, so liver biopsies are required.

CHB proceeds through phases with a primary distinguishing feature being the presence of hepatitis B e antigen (HBeAg), which usually represents higher levels of HBV replication. *S* is transcribed primarily from cccDNA in HBeAg-positive CHB and from iDNA in HBeAg-negative CHB (2–4). However, in both HBeAg-positive and -negative CHB, we and others have reported that NUC treatment is associated with transcriptional silencing of cccDNA as well as a decrease in the overall number of infected hepatocytes (5–10). Although a decline in blood HBsAg would be expected with cccDNA transcriptional silencing and reductions in the number of infected cells, most people with CHB taking NUCs do not show substantial declines in HBsAg levels. Indeed, HBsAg loss during NUCs is rare (11, 12). These paradoxes strongly indicate that *S* transcripts from iDNA along with persistent low-level *S* transcription from cccDNA may maintain production of HBsAg during NUCs. This hypothesis is supported by our previous study of bulk liver from people with HIV and CHB. Individuals with chiefly cccDNA-derived transcription were more likely to have declines in blood HBsAg levels during NUCs compared with people with chiefly iDNA-derived transcription (2). To better understand the cellular and molecular dynamics underlying HBsAg production during NUCs, we used single-cell laser capture microdissection (scLCM) and droplet digital PCR (ddPCR) on paired biopsies from individuals with HIV and CHB who received varying durations of NUC treatment. We linked our findings to viral antigens in liver and serological testing of distinct HBsAg isoforms in blood that are predicted to be differentially present during chiefly cccDNA- versus iDNA-derived transcription.

## Results

### Study participants.

All 10 participants were men, with a median age of 49 years at biopsy 1 (Table 1). Participants were stratified by the duration of NUC exposure at biopsy 1 into an early group and a prolonged group. In the 5 early group participants, the median NUC duration was 3 weeks (range 0–1.5 years) prior to biopsy 1 with a median (range) plasma HBV DNA level of 6.6 (4.2–8.6) log_10_ IU/mL that declined to 1.6 (undetectable to 2.9) log_10_ IU/mL at biopsy 2, which was obtained a median of 3.7 years after biopsy 1. All 5 of the early group participants had a maximum decline in blood quantitative HBsAg (qHBsAg) of ≥0.5 log_10_ IU/mL during the study period relative to the level at biopsy 1 (median [range] decline 1.2 [0.5–2.0] log_10_ IU/mL) (Table 1 and Supplemental Figure 2; supplemental material available online with this article; https://doi.org/10.1172/JCI184243DS1). Four participants in this group were HBeAg positive throughout the study, while 1 participant seroconverted to HBeAg negative shortly after biopsy 1. The 5 participants in the prolonged group had received NUCs for a median (range) of 7 (5 to 8) years prior to biopsy 1. All except 1 participant had undetectable HBV DNA at both biopsies and all had less than 0.5 log_10_ IU/mL decline in qHBsAg during the study period relative to the level at biopsy 1 (Table 1 and Supplemental Figures 2 and 3). Four of those participants were HBeAg negative and 1 was HBeAg positive throughout.

### cccDNA-derived transcription diminishes during NUCs.

**Figure 1:**
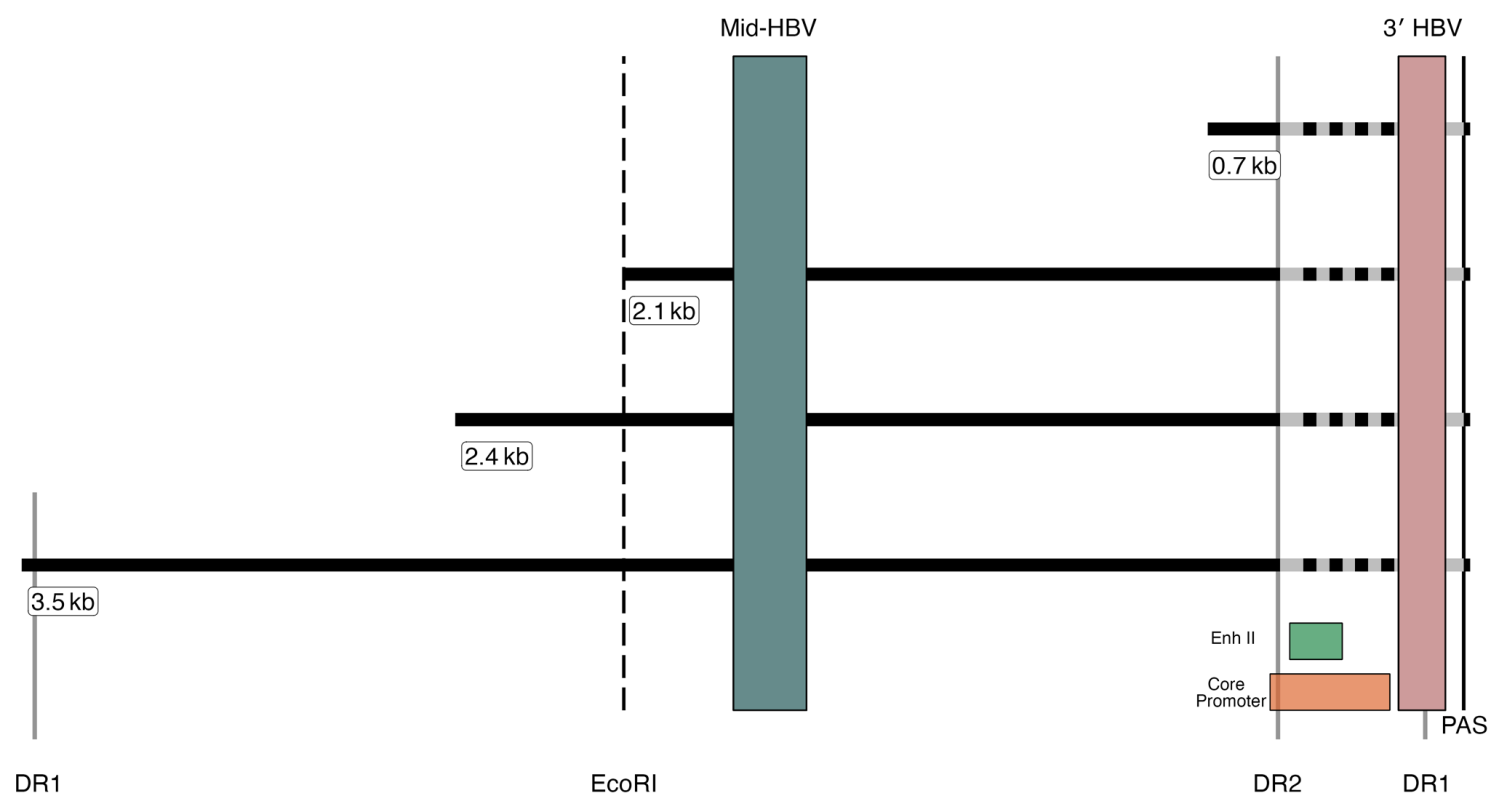
Transcriptional map with human-virus chimeric breakpoints from liver tissue. Horizontal lines depict the 4 canonical HBV mRNAs produced by each of the open reading frames. The variable chimeric virus-host regions are displayed as hashed lines at the 3′ end of transcripts. Solid vertical lines show the positions of DR2, DR1, and the canonical polyadenylation signal (PAS), respectively. The colored bars depict the 2 ddPCR target amplicons: mid-HBV and 3′-HBV. The locations of the HBV enhancer II (EnhII) and core promoter regions are displayed as green and orange boxes, respectively. The dashed line represents the EcoRI cut site, which is included for reference.

To understand changes in HBV transcription during NUCs in both groups, we first determined the proportion of cells with HBV transcripts in biopsies 1 and 2 from each participant by applying our scLCM/multiplex ddPCR (see Methods) approach to single-cell equivalents. Our multiplex ddPCR assay targets 2 regions of the HBV transcriptome: (a) the middle of the *S* gene (mid-HBV), which is present in both cccDNA- and iDNA-derived transcripts and captures transcripts that are translated into HBsAg, as well as longer transcripts (e.g., pregenomic RNA), and (b) the 3′ terminus of cccDNA-derived transcripts (3′-HBV) that is absent in iDNA-derived transcripts because these have HBV-human hybrid chimeric junctions that lie in the domain between DR2 and the 3′ canonical polyadenylation signal (PAS) (Figure 1) (4). This strategy is supported by previously reported transcription maps and our own sequencing demonstrating that the vast majority of HBV-human hybrid junctions are indeed located upstream of our 3′-HBV assay, and therefore would not be detected by the assay (Supplemental Figure 1 and Supplemental Methods).

**Figure 2:**
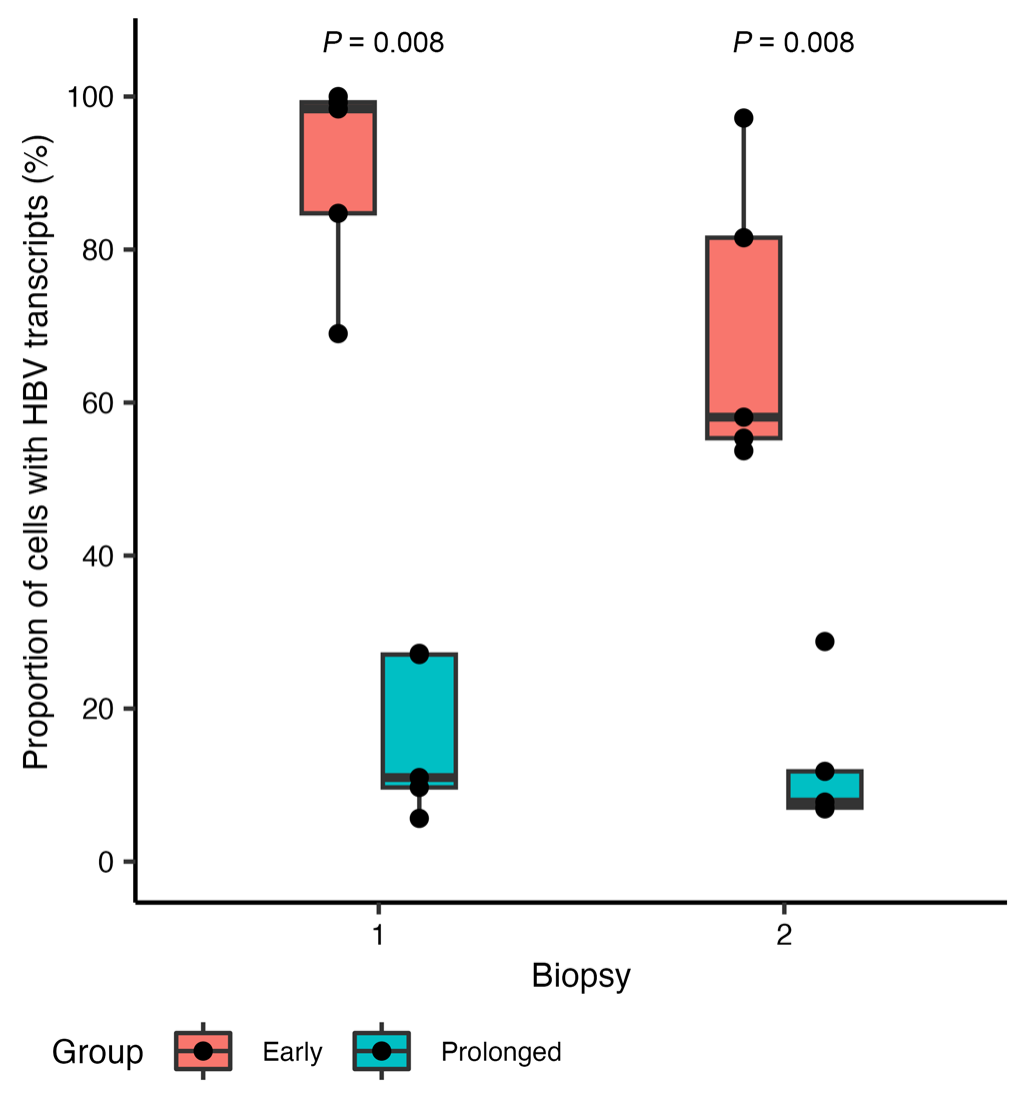
Lower proportion of cells with HBV transcripts with prolonged NUC treatment. The proportion of all analyzed cells with detectable transcripts (positive for mid-HBV, 3′-HBV, or both) in each biopsy is shown; each dot represents a person at their respective biopsies. The boxes span the first and third quartiles with a horizontal line representing the median. The tails correspond to the minimum and maximum of that respective group. Wilcoxon’s rank-sum test was used to compare between groups. Red, early group; blue, prolonged group.

After discarding cell fragments that did not meet our quality control, we examined a median (range) of 176 (85 to 271) cells in each biopsy, totaling 3,436 cells (see Methods). The proportion of cells with HBV transcripts (mid-HBV, 3′-HBV, or both) at biopsy 1 was higher in the early than in the prolonged participant group (median 98% vs. 11%, *P* = 0.008) (Figure 2). In the early group, at biopsy 2, after a median 3.7 years on NUCs, these proportions declined in all participants, with one participant’s declining by 45%. In contrast, in the prolonged group, the proportions remained stable. Notably, the overall decrease in proportions of cells containing HBV transcripts in the early group by biopsy 2 made them appear to be more similar to the prolonged group at biopsy 1, although these proportions remained significantly greater in the early group at biopsy 2 compared with the prolonged group at biopsy 1 (median 7 years on NUCs) (median 58% vs. 11%) (*P* = 0.008). This result may reflect the longer duration on NUCs in the prolonged group biopsy 1 than in the early group biopsy 2, but may also have a contribution from HBeAg status.

After characterizing changes in proportions of cells with HBV transcription during NUCs, we next focused on understanding whether there was a change in the abundance of viral transcripts (i.e., the mid-HBV and 3′-HBV transcripts) in actively transcribing individual cells between biopsies, as we have previously shown for pregenomic RNA (5–7). Thus, for each cell studied in each biopsy, we determined the quantity of the mid-HBV and 3′-HBV transcripts and calculated a median value per biopsy. In the early group participants, these values for mid-HBV quantities per cell decreased from a median (range) of 56 (4 to 232) copies per cell to 8 (0 to 92) copies per cell between biopsies 1 and 2 (*P* < 10^–5^) (Supplemental Figure 4). Similarly, we also found that cccDNA-derived transcripts, as defined by the 3′-HBV amplicon, decreased from a median (range) of 112 (8 to 200) copies per cell at biopsy 1 to 0 (0 to 136) copies per cell at biopsy 2 (*P* < 10^–5^) (Supplemental Figure 5). In contrast, in the prolonged group, the amount of HBV transcription per cell was low at both biopsies: the median was below detection for both the mid-HBV and 3′-HBV amplicons; therefore, at least 50% of cells did not contain these transcripts in any of the prolonged group participant biopsies. Because we tested hundreds of cells in each individual, we could still observe that the quantity per cell of the mid-HBV amplicon exhibited a small but significant decline (*P* = 0.0001) in the prolonged group that was not seen with the 3′-HBV amplicon (*P* = 0.2). Taken together, these data suggest that changes in HBsAg during the first few years of treatment are likely due to both a decrease in the number of infected cells and a downregulation of cccDNA-derived transcription in individual cells.

### iDNA-derived transcription is maintained during NUCs.

Since cccDNA transcription is downregulated and the number of infected hepatocytes decreases during NUCs, we tested the hypothesis that iDNA transcription continues contributing to the maintenance of circulating HBsAg during NUCs. To test this hypothesis, we focused on cells actively transcribing *S* in each biopsy and classified each cell as having chiefly cccDNA-derived, iDNA-derived, or mixed (both cccDNA- and iDNA-derived) transcription, as determined by the iDNA transcriptional index (iDNA-TI) (see Methods) (2). The iDNA-TI is the ratio of the quantities of mid-HBV to 3′-HBV amplicons: a value ≤ 1 indicates chiefly cccDNA-derived transcription, >1 indicates mixed transcription, and only mid-HBV transcripts (no detectable 3′-HBV transcripts) indicates chiefly iDNA-derived transcription.

**Figure 3:**
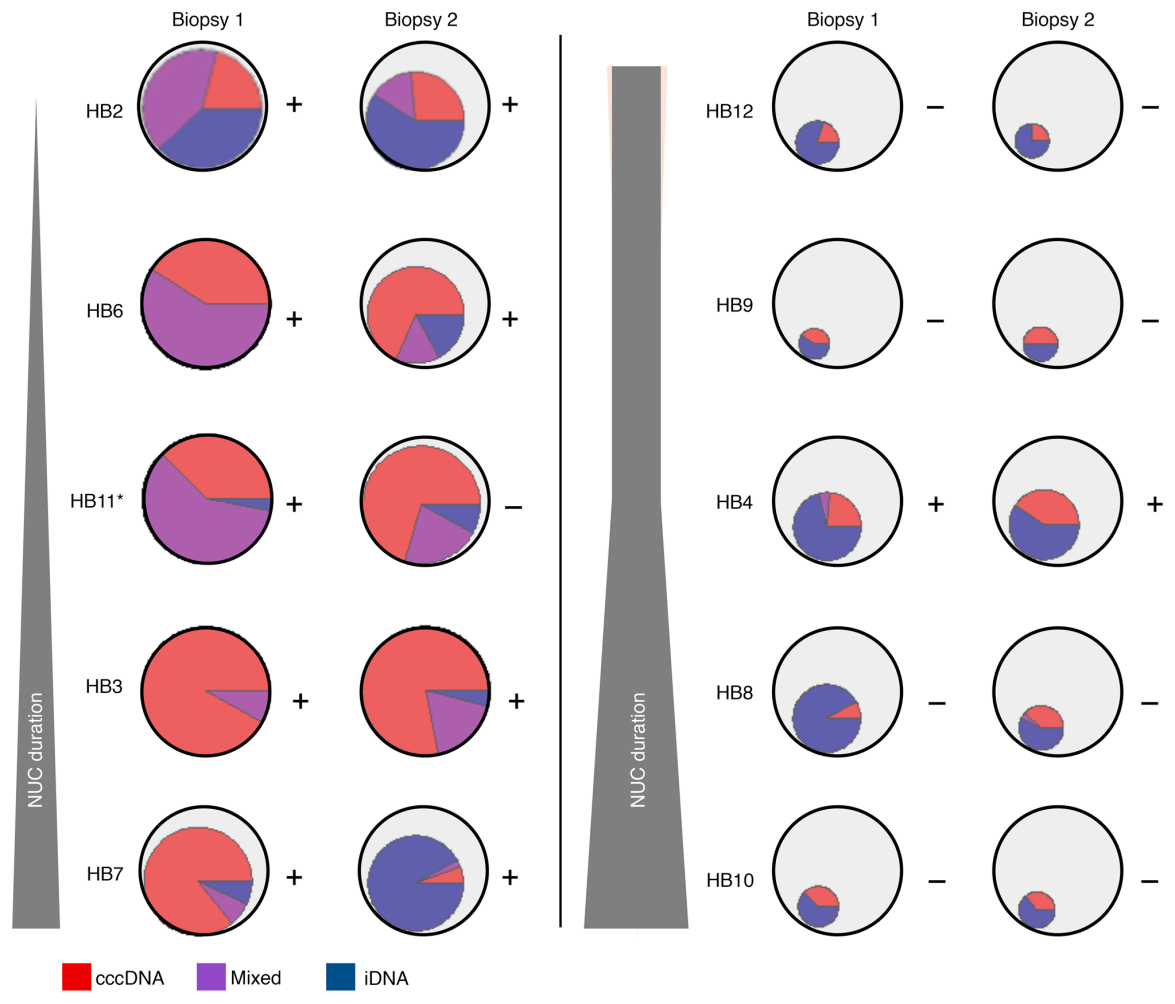
Changes in the cellular source of HBV transcription in paired biopsies during early and prolonged NUC treatment. Shown are the proportion of hepatocytes that are transcriptionally active (i.e., contain viral transcripts). The larger circles, outlined in black, are fixed in size. The inner inscribed pie charts depict the proportion of hepatocytes that were found to be transcriptionally active. When the inner pie chart fills the entire larger circle, it indicates that 100% of hepatocytes are transcriptionally active, whereas smaller proportions are denoted by their respective areas. Each pie chart is subdivided by color to indicate the proportion of transcriptionally active cells that have either chiefly cccDNA-derived transcription (red), chiefly iDNA-derived transcription (blue), or mixed transcription (purple). HBeAg status (positive or negative) at the time of each biopsy is indicated to the right of each circle. Participants are ordered from top to bottom by the duration of NUC therapy prior to biopsy 1, as indicated by the vertical gray wedges to the left of all participants. *P* values for each change are shown in Supplemental Table 1. HB11* indicates the individual who seroconverted HBeAg between biopsies.

Using this index, we determined the proportion of cells actively transcribing *S* with chiefly cccDNA-derived, iDNA-derived, or mixed transcription in each biopsy. Interestingly, in the early group, the proportion with chiefly iDNA-derived transcription was enriched in every participant, ranging from a 5% (HB11) to an 85% (HB7) increase, from biopsy 1 to biopsy 2 (Figure 3, blue; *P* < 0.05 for each participant; Supplemental Table 1). In contrast, cells with mixed transcription declined in 4 of 5 participants in the early group by 5% (HB7) to 44% (HB6) from biopsy 1 to biopsy 2 (Figure 3, purple; *P* < 0.05 for all except HB7; Supplemental Table 1). Whereas the total number of cells actively transcribing *S* decreased during NUCs (Figure 3, smaller inner circle), the proportion of these cells with chiefly cccDNA-derived transcription was more variable across individuals: 2 showed a decrease (HB3 by 14% and HB7 by 80%) and the other 3 showed increases (HB2 by 5%, HB6 by 27%, and HB11 by 33%). However, for 2 of the participants, HB2 and HB6, when the total cell population was studied, the change in cccDNA-derived transcription between biopsies was nearly negligible because of the decrease in the number of infected cells. These data support that in the approximately 3–4 years between biopsies in the early group, cells with primarily iDNA-derived transcription were likely enriched from the diminished pool of hepatocytes with mixed transcription. In other words, early after NUCs, cells with mixed transcription from both iDNA and cccDNA undergo cccDNA transcriptional silencing during NUCs, yielding viral transcripts that are chiefly iDNA derived.

This shift had already occurred in the prolonged group biopsies, where fewer cells were transcriptionally active and per-cell transcription was dampened overall in comparison with early group participants. Further, at both biopsies in the prolonged group, very few cells appeared to have mixed transcription (median of 0% in both biopsies), supporting our findings from the early group that cells with mixed transcription declined with NUCs (Figure 3). Notably, the proportion of cells with chiefly iDNA-derived transcription exceeded that of cells with chiefly cccDNA-derived transcription in all prolonged group participants at both biopsies. The proportion of transcriptionally active cells that were classified as chiefly iDNA derived declined numerically in all participants, ranging from 5% (HB12) to 35% (HB8) (but this was only significant in HB8, *P* = 0.002, with *P* > 0.05 for others; Supplemental Table 1), from biopsy 1 to biopsy 2, indicating that iDNA-derived transcription decays slowly if at all with long-term NUCs.

In the prolonged group participants, chiefly cccDNA-derived cells were minimally enriched from biopsy 1 to biopsy 2, and this was only significant in HB8 (Supplemental Table 1). It is important to note that both of these changes were minimal in the context of the total cell population, and likely reflected relative changes between cell type rather than increases in total numbers of chiefly cccDNA-derived cells. When summarizing the early and the prolonged NUC groups, we observed that as the total number of actively transcribing cells diminished, the chiefly iDNA-derived cell component took an increasing fraction of actively transcribing cells (Figure 3). While chiefly iDNA-derived cells were expected in the prolonged NUC group since they are mostly HBeAg negative, it was also true of the early NUC group at biopsy 2, who mostly remained HBeAg positive at biopsy 2. Thus, cells with iDNA-derived transcription become dominant during prolonged NUCs, accounting for the majority of the continued HBsAg despite years of antiviral treatment. We also note that, irrespective of HBeAg status and duration of NUCs, cccDNA-derived transcription persists at low levels in all people.

The transition from chiefly cccDNA-derived to chiefly iDNA-derived transcription is best exemplified by the early group participant (HB7) who was on NUCs the longest at biopsy 1 (1.5 years). The participant had a nearly complete transition from chiefly cccDNA-derived to chiefly iDNA-derived transcription between biopsies (7/98 [7.1%] to 80/87 [92%] cells with chiefly iDNA-derived transcription at biopsies 1 and 2, respectively, *P* < 0.0001). By biopsy 2, HB7 resembled people in the prolonged group in terms of both the source of viral transcription and the duration of NUC therapy, although HB7 did not have a change in HBeAg status. Demonstrating this shift in HB7, the median (range) abundance of the mid-HBV amplicon was similar between biopsies (4 [0 to 457] and 8 [0 to 120] copies per cell at biopsies 1 and 2, respectively) while the median (range) abundance of 3′-HBV declined from 8 (0 to 955) copies per cell to 0 (0 to 16) copies per cell, consistent with a reduction in cccDNA-derived transcription but maintenance of iDNA-derived transcription (Supplemental Figures 4 and 5).

### Modeling decay of transcriptionally active cells.

**Figure 4:**
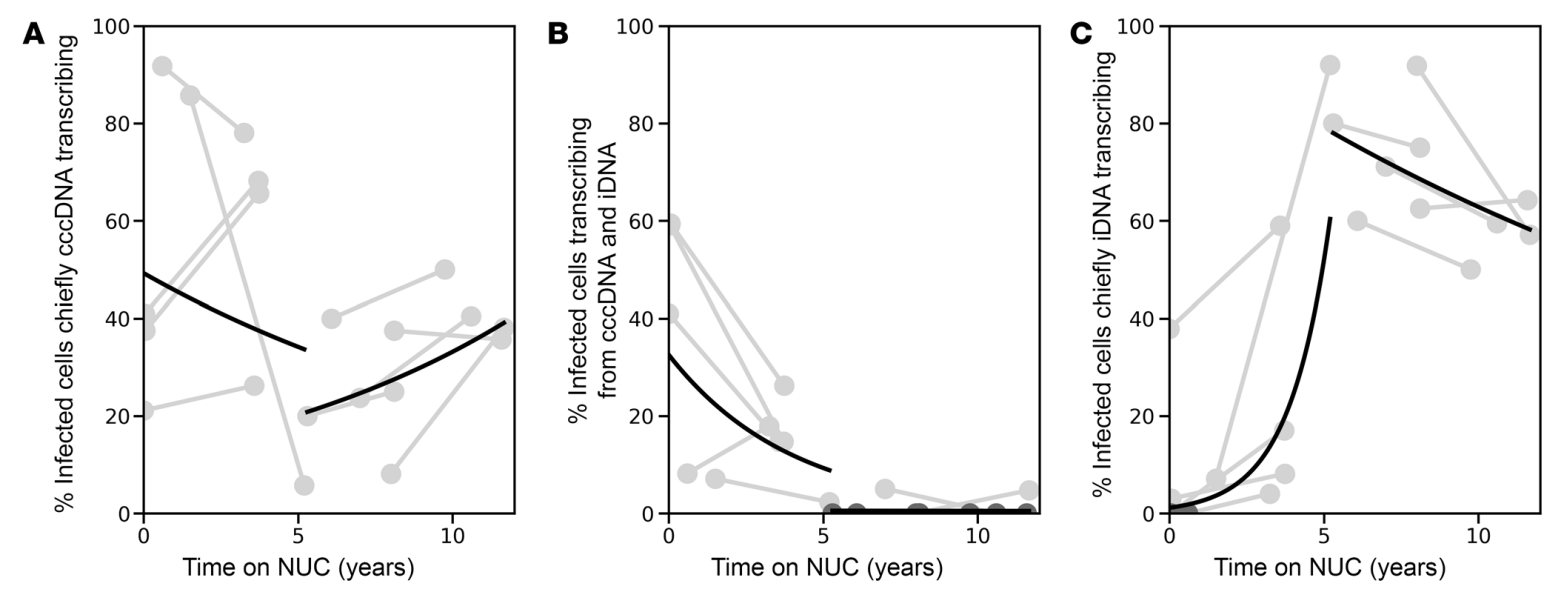
Among cells actively transcribing viral RNA there are different decay rates depending on the transcriptional source. Each gray line represents a participant, with points corresponding to biopsies 1 and 2. The time on NUCs (years) is plotted against the proportion of cells actively transcribing *S* chiefly from cccDNA (iDNA-TI ≤ 1) (**A**), from both cccDNA and iDNA (iDNA-TI > 1) (**B**), and chiefly from iDNA (only mid-HBV positive) (**C**). Mixed-effects modeling was used to generate rates across the group, represented by the black lines (see Methods).

Focusing on cells actively transcribing *S*, we used our single-cell data to develop mathematical models describing the decay of the fractions of cells transcribing *S* from chiefly cccDNA, mixed, or chiefly iDNA. We evaluated multiple models to describe empirically the dynamics of these different cell populations (see Methods), where the decay results from a balance of production of infected cells (new infections or cell division) and loss of infected cells (e.g., cell death). We found that the observed decays were best fit by independent and different rates in the early and prolonged groups. That is, the decay observed in the prolonged group was not consistent with long-term continuation of the decay seen in the early group. The most salient features were an initial slow decline (half-life of ~10 years) followed by an unexpected slow increase in the fraction of cells actively transcribing *S* from chiefly cccDNA at late times (doubling time of 7 years) (Figure 4A). It is worth noting that the large variability in the data for the initial slow decline makes the estimated half-life less precise. We also observed a rapid decay in cells with mixed transcription in the early group (half-life of 2.7 years) followed by a near loss in their detection in the prolonged group (Figure 4B). This was accompanied by a fast increase in the early group in the fraction of cells actively transcribing *S* from chiefly iDNA, with a doubling time of about 1 year (Figure 4C). These findings were substantiated by modeling of the decay of the fraction of total cells exhibiting any 3′-HBV amplicon or any mid-HBV amplicon. In the early group, the fraction of cells expressing the mid-HBV amplicon had a half-life of 5 years, which slowed down slightly to about 7.8 years in the prolonged group (Supplemental Figure 6B). On the other hand, the half-life of cells with 3′-HBV amplicons mirrored that of the mixed population, 2.7 years. In contrast, there was essentially no decay of the fraction of cells with 3′-HBV amplicons in the prolonged group; indeed, we observe that these cells had a slow but non-significant increase (doubling time of 9.2 years) (Supplemental Figure 6A), which may be a consequence of ongoing cccDNA transcription resulting in a low level of ongoing infection (Figure 3). Taken together with the other decay rates, the observed increase in the fraction of cells with chiefly iDNA-derived transcription is likely due to a decrease in the population of cells with mixed transcription: as cells in the mixed population undergo cccDNA transcriptional silencing, these cells chiefly transcribe from iDNA.

### Linking viral protein production in liver to the source of viral transcription.

**Figure 5:**
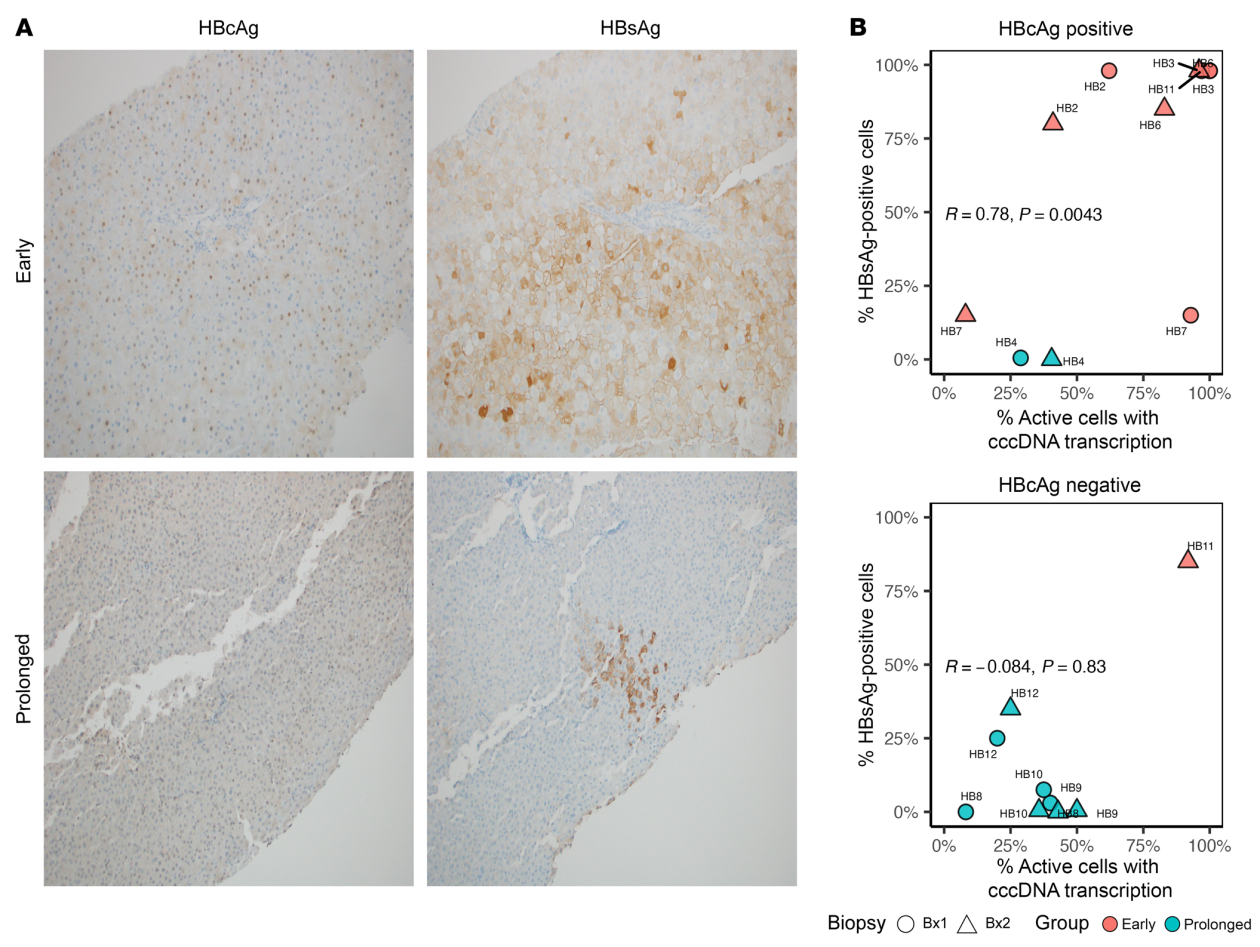
cccDNA-derived transcription drives intrahepatic viral antigen production early after NUCs but not after prolonged NUCs. Immunohistochemistry was used to stain for hepatitis B core antigen (HBcAg) and hepatitis B surface antigen (HBsAg) in each biopsy, and the amount of staining was quantified by a pathologist who was blinded to participant identity. (**A**) Representative HBcAg (left) and HBsAg (right) staining images at biopsy 1 for 1 participant in the early treatment group, HB6 (top), and 1 in the prolonged group, HB12 (bottom). Original magnification, ×200 (top images) and ×100 (bottom images). (**B**) After stratifying by HBcAg-positive biopsies (top) and HBcAg-negative biopsies (bottom), we correlated the percentage of transcriptionally active cells with cccDNA-derived transcripts (including chiefly cccDNA and mixed) with the percentage of cells positive for HBsAg staining. Red and blue dots (biopsy 1) represent early and prolonged individuals, respectively. Spearman’s correlation coefficients and associated *P* values are shown. Bx, biopsy.

With an understanding of changes in HBV transcription in hepatocytes, we next sought to reconcile HBsAg protein production with the source of viral transcription, since historically immunohistochemistry of HBsAg correlated inconsistently with HBV replication (13). Transcriptional maps reported previously indicate that the full suite of viral proteins is not likely to be produced from iDNA (14, 15) even though rare integrations contain nearly the entire coding sequence of the HBV genome (Figure 1), especially because the genomic organization of the circular HBV genome is disrupted in the linear iDNA. Specifically, the promoter and enhancer regions for the core gene are separated from the core gene by HBV-human chimeric junctions (Figure 1 and Supplemental Figure 1). Therefore, because the core protein is predicted to be largely derived from cccDNA, we hypothesized that viral transcription and core protein production are correlated during cccDNA-derived but not during iDNA-derived transcription. To test this, we predicted that the proportion of cells with positive HBsAg staining by immunohistochemistry would correlate with the proportion of cells with any cccDNA-derived transcription only in tissues that were positive for hepatitis B core antigen (HBcAg) by immunohistochemistry. Of the 20 biopsies, 11 had detectable HBcAg, which included all of the early group and 1 prolonged group participant (Figure 5A). As expected, we observed a correlation in HBcAg-positive biopsies between the proportion of transcriptionally active cells with any evidence of cccDNA-derived transcription and the percentage of cells positive for HBsAg staining (*R*^2^ = 0.78, *P* = 0.004). In contrast, among HBcAg-negative biopsies, there was no observable association (*R*^2^ = –0.084, *P* = 0.83) (Figure 5B).

### Source of transcription affects changes in blood viral markers.

**Figure 6:**
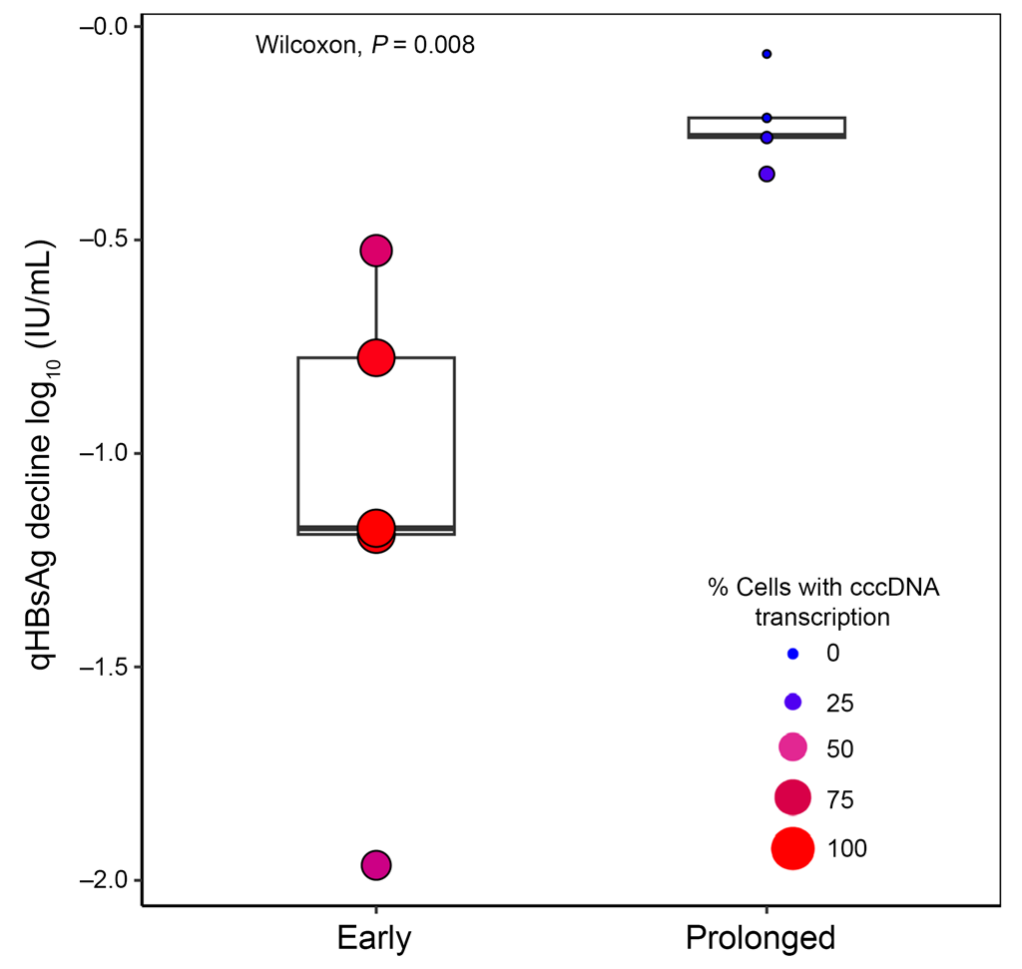
Participants with higher proportions of cells with cccDNA-derived transcription at biopsy 1 had larger declines in blood qHBsAg with NUC treatment. The *y* axis represents the maximum decline of qHBsAg after NUCs relative to measures taken at biopsy 1 (Supplemental Figure 2), while the size and color of the points indicate the proportion of all cells with any cccDNA transcription (including chiefly cccDNA and mixed) at biopsy 1. The boxes span the first and third quartiles, and the horizontal lines represent the median. The tails correspond to the minimum and maximum of that respective group. Wilcoxon’s rank-sum test was used to compare qHBsAg declines between early and prolonged groups.

We next investigated whether peripheral blood markers can provide insight into the intrahepatic source of transcription. In this and prior studies, we and others have shown that NUCs are associated with silencing of cccDNA transcription but not iDNA transcription (5–10). Therefore, we hypothesized that there would be a greater decline in blood qHBsAg between biopsies when most cells at biopsy 1 have cccDNA-derived rather than iDNA-derived transcription. To test this hypothesis, we determined the maximum qHBsAg decline between biopsy 1 and any point after biopsy 1. Among the early group, a median (range) of 96% (53%–100%) of cells had some cccDNA-derived transcription at biopsy 1; these participants had a median (range) of 1.2 log_10_ (0.5–2.0) IU/mL reduction in qHBsAg. In contrast, among prolonged group participants, the median (range) proportion of cells with cccDNA-derived transcription was 2.3% (2.2%–7.8%) at biopsy 1, and qHBsAg values did not change appreciably (median [range] 0.3 [0.06–0.35] log_10_ IU/mL decline) (Figure 6). These results demonstrate that when most cells exhibit cccDNA-derived transcription, qHBsAg levels decline with NUC treatment.

We also separately explored whether HBsAg isoforms in blood were correlated with the source of transcription. Transcription maps from HBeAg-negative individuals, who transcribe chiefly from iDNA, suggest that the region encoding large HBsAg (L-HBsAg) is integrated less frequently than those encoding middle HBsAg (M-HBsAg) and small HBsAg (Sm-HBsAg) (Figure 1) (4), and its promoter may have greater dependence on EnhII than the promoters for the latter isoforms (16). Thus, we predicted that people with chiefly cccDNA-derived transcription would be more likely to produce L-HBsAg than people with chiefly iDNA-derived transcription. Supporting this hypothesis, we found that the proportion of transcriptionally active cells with any cccDNA-derived transcription, as determined by our iDNA-TI, correlated with L-HBsAg quantities in blood at time of biopsy (*R*^2^ = 0.66, *P* = 0.004) (Supplemental Figure 7, A and B).

Conversely, we tested whether the proportion of HBsAg that is Sm-HBsAg could be inferred from the proportion of cells with cccDNA- versus iDNA-derived transcription. Since our assay did not directly measure Sm-HBsAg and because L-HBsAg constituted a small proportion of the HBsAg isoforms, we estimated the relative proportion of Sm-HBsAg by determining the ratio of M-HBsAg to total-HBsAg (T-HBsAg, which captures all isoforms) such that a higher M-HBsAg/T-HBsAg (M/T) ratio corresponds to lower proportions of Sm-HBsAg (17, 18). We found that the early group had a lower proportion of Sm-HBsAg compared with the prolonged group (*P* = 0.03) (Supplemental Figure 8A). Furthermore, liver biopsies with chiefly iDNA-derived transcription correlated inversely with the M/T ratio (*R*^2^ = –0.66, *P* = 0.003), consistent with higher Sm-HBsAg proportions in blood at the time of biopsy in people with chiefly iDNA-derived transcription (Supplemental Figure 8B). Taken together, these observations suggest that in people with prolonged NUCs, more of the circulating HBsAg is made up of Sm-HBsAg, which may be driven by iDNA-derived transcription. Collectively, our results support that decreases in the number of cells with cccDNA-derived transcription during NUCs are associated with decreases both in qHBsAg and in L- compared with Sm-HBsAg.

## Discussion

Applying high-resolution molecular tools to paired liver biopsies from 10 people coinfected with HIV and HBV, we demonstrate that with longer NUC duration *S* transcripts are maintained primarily by iDNA transcription albeit with a small but persistent contribution from cccDNA; this was observed irrespective of HBeAg status. Early NUC exposure in people with chiefly cccDNA-derived transcription (who were HBeAg positive) was associated with a decline in blood HBsAg levels. Further, whereas people with chiefly cccDNA-derived transcription had detectable viral antigens in liver and blood, these were not well detected in people with chiefly iDNA-derived transcription. Although participants with prolonged NUC exposure had fewer transcriptionally active cells than those with early NUC exposure, a higher proportion of these transcriptionally active cells in the prolonged group had iDNA-derived rather than cccDNA-derived transcription. Consistent with this, among transcriptionally active cells, we observed a multiphase decay showing a slow decline in cells with predominantly cccDNA-derived transcription and a relatively fast increase in cells with iDNA-derived *S*. Most importantly, participants with mainly iDNA-derived transcription at biopsy 1 had minimal changes in blood qHBsAg with continued NUC treatment, consistent with the lack of predicted activity of NUCs on iDNA transcription.

NUCs prevent new infection events, resulting in a diminished number of infected cells, but they are also associated with cccDNA transcriptional silencing (5–10). Although the precise mechanism of NUC-associated cccDNA transcriptional silencing has yet to be determined, it may be due to host factors that target cccDNA, mutations induced in the viral genome, epigenetic changes, or some combination of these. Thus, it has been hypothesized that blood HBsAg during prolonged NUCs is maintained primarily by iDNA-derived HBsAg. In this study, we uncovered the single-cell composition of HBV-infected hepatocytes that produce and maintain HBsAg levels during NUCs; we observed a global transition from primarily cccDNA-derived to primarily iDNA-derived transcription with longer NUC duration. Whereas the prolonged group participants were largely HBeAg negative, this transition occurred even in the early group participants who did not seroconvert their HBeAg status. It seems likely that this transition is due to cccDNA-derived transcription dominating *S* production until either the majority of the cccDNA is silenced or those hepatocytes decay during NUCs, yielding detectable iDNA *S* transcription. This is also consistent with the near disappearance of cells with mixed transcription in prolonged treated individuals; we hypothesize that the cccDNA in cells with mixed transcription is progressively silenced, yielding a greater proportion of cells that have chiefly iDNA-derived transcription that enrich that population. It is also notable that in the prolonged group the proportions of hepatocytes with either iDNA-derived or cccDNA-derived transcription were relatively stable, suggesting either that these cells evade immune or senescent clearance as a result of inadequate antigen presentation or that low-level replication maintains these cells. It is also important to note that residual cccDNA transcription with prolonged NUC treatment may replenish both cccDNA and iDNA in hepatocytes. Thus, in prolonged NUC treatment, HBsAg levels are a result of the balance between the natural decay of hepatocytes and replenishment of cccDNA and iDNA.

Our modeling revealed multiphase decays that are consistent with several subpopulations of infected cells decaying at different rates. We found evidence of 2 populations of cells with cccDNA transcription. The first, containing cells transcribing from both cccDNA and iDNA (mixed), decayed relatively quickly when NUCs were started (half-life = 2.7 years) and largely disappeared with longer NUCs. The second population, consisting of cells transcribing chiefly from cccDNA, also initially decayed when NUCs were started but then were maintained with longer NUC duration and, surprisingly, showed slower decay than cells with chiefly iDNA-derived transcription. This longer-lived subpopulation of chiefly cccDNA-transcribing cells that persists after years of NUCs is consistent with a reservoir of infection that has persistent, low-level transcription, which is also supported by another study demonstrating ongoing evolution of HBV in people with suppressed viremia due to long-term NUCs (19). Another possible explanation for the apparent slower decay of chiefly cccDNA-transcribing cells compared with mixed cells arises if cccDNA transcriptional silencing is incomplete; in other words, when cccDNA is partially silenced in mixed cells, they become cells with chiefly iDNA-derived transcription, whereas when cells with chiefly cccDNA-derived transcription are partially silenced, they still become cells with chiefly cccDNA-derived transcription but with a lesser magnitude of viral transcription. A third population of cells that we found were those with chiefly iDNA-derived transcription that increased quickly as a proportion of transcriptionally active cells after NUC initiation, and then showed slow decay with longer NUC duration. Taken together, these results are consistent with a loss in cells with chiefly cccDNA-derived transcription early during NUCs due to (a) partial prevention of new infections and (b) cccDNA transcriptional silencing. This is followed by a natural slow decline of all infected cells due to cell death. Since cells with chiefly iDNA-derived transcription were largely found in the absence of viral antigens such as HBcAg, this slowly decaying population may represent infected cells that present a restricted suite of viral antigens, allowing them an added measure of evasion from immune surveillance (20). Although multiphase decay supports the hypothesis of distinct subpopulations of infected hepatocytes decaying at different rates, it is possible that NUC-associated transcriptional silencing of cccDNA leads to changes (i.e., increase) in the half-life of cells that contain viral genomes. An alternate explanation for differential decay may be differences in hepatocyte renewal mechanisms. One study demonstrated that higher-ploidy hepatocytes are less likely to divide into daughter cells, thus allowing multinucleated hepatocytes to persist longer (21). These infected cells may be a long-lived population thus serving as a quiescent reservoir for ongoing replication. Since repeated mitosis of cccDNA-containing hepatocytes has been suggested as a potential strategy for cccDNA clearance, a slower rate of cell division in a subpopulation of cells containing cccDNA may explain its long-term persistence (20, 22).

There has been great interest in understanding how intrahepatic markers of transcription are reflected in peripheral blood biomarkers, particularly HBsAg, since functional cure requires its loss. Earlier studies found that blood qHBsAg correlated well with intrahepatic cccDNA levels before treatment initiation, but these parameters correlated poorly after extended NUCs (11, 13, 23, 24). Our findings help to explain the lack of correlation between measures of HBV replication that center on cccDNA quantities and blood qHBsAg levels in people taking prolonged NUCs, since cccDNA in this scenario is largely transcriptionally silent while HBsAg is mostly derived from iDNA. We extended these observations by demonstrating that in participants with a high proportion of cells with cccDNA-derived transcription, there was a decline in qHBsAg with NUCs, whereas that was not the case in participants with chiefly iDNA-derived transcription. This is consistent with other studies showing that the presence of integrated *S* at baseline in people who are NUC-naive was associated with poorer responses in qHBsAg decline (25, 26). Even when transcriptionally active integrations decrease during NUC therapy, the observation remains that qHBsAg is stable when iDNA transcription predominates (Supplemental Figure 1) (27, 28). We also demonstrate that HBsAg isoforms may reflect intrahepatic HBV transcription, since the proportion of cells transcribing from cccDNA correlated with amounts of L-HBsAg in blood. Similarly, qHBsAg decline during NUCs, which is greater with cccDNA transcription, was also greater in people with more L-HBsAg. Conversely, though iDNA effectively transcribes PreS2/S, the proportion of Sm-HBsAg appeared higher in the prolonged group than in the early group. Pfefferkorn et al. found that in people who had HBsAg loss with NUCs, L- and M-HBsAg declined before total HBsAg, which may reflect the silencing or elimination of hepatocytes with chiefly cccDNA-derived transcription (29). Thus, we present mechanistic and observational support that blood biomarkers reveal clues about intrahepatic transcription, although further work is needed to validate these as clinically useful biomarkers and to potentially explore others.

We encountered several challenges during this study. First, despite the intensive single-cell investigation, the core biopsy is a fraction of the whole liver, so there is the possibility of sampling error. It is encouraging that our findings correlate closely with blood markers and with liver tissue staining, supporting that the cells were representative of the liver. A second limitation is that these individuals were all from one geographic region, men, and with HIV receiving tenofovir disoproxil fumarate. Single-cell studies will need to be expanded into other geographic regions, women, people on entecavir, and people without HIV to confirm our findings. A third limitation is that we focused on active transcription by studying cccDNA-derived and iDNA-derived transcripts rather than the total number of cccDNA molecules or integrations. While we have reported on the former earlier, the absence of direct measurements of iDNA was intentional, since our aim was to explain the persistence of HBsAg, which must involve transcriptionally active iDNA. It is possible that the dynamics of total integrations, many of which might be transcriptionally inactive, may be distinct compared with the dynamics of only the transcriptionally active iDNA examined here (30). A fourth limitation is that heterogeneity exists regarding the timing of NUC initiation relative to biopsy 1 and NUC adherence between biopsies in the early group (Supplemental Figure 3). However, despite this, *S* transcription is dominated by cccDNA early during NUCs. A fifth limitation is that our modeling was based on 5 individuals in both the early and prolonged groups, which is partially mitigated by the large number of cells studied. Lastly, all of the early group participants were HBeAg positive and only 1 prolonged group participant was HBeAg positive, so our conclusions about NUC duration enhancing iDNA-derived HBsAg cannot be fully distinguished from HBeAg-negative status. Overall, we observed that by biopsy 2, the early group proportions of transcriptionally active cells began to resemble those at biopsy 1 from the prolonged group. This was best exemplified by HB7 in the early group, who had been on NUCs the longest and had the same transcriptional characteristics at biopsy 2 as the HBeAg-negative biopsies in that group by single-cell analysis, supporting our conjecture that increasing time on NUCs enhances iDNA-derived HBsAg irrespective of HBeAg status. Nevertheless, it will be important to verify our findings in more people with CHB at different stages of disease and different durations of treatment.

By focusing on single HBV-infected hepatocytes as the unit of functional cure, we offer a granular understanding of how the source of *S* transcription changes from cccDNA to iDNA during NUC treatment. We further show that, consistent with the shift toward iDNA-derived transcription, there are decreases in cccDNA-derived viral antigens such as intrahepatic HBcAg and blood L-HBsAg during prolonged NUC treatment, offering insights about these markers in people with chiefly cccDNA-derived but not iDNA-derived transcription. Importantly, during long-term NUC therapy, we still found occasional cells with low levels of cccDNA-derived transcription that persisted. Modeling revealed that achieving a functional cure will require addressing the slowly decaying iDNA-derived *S* transcripts and permanently silencing cccDNA transcription. Moreover, clinical trials of existing and emerging agents should incorporate liver biopsies to understand how the complex viral reservoir of infected hepatocytes responds to treatment.

## Methods

### Sex as a biological variable.

Our study exclusively examined men because samples from women were not available. We do not expect these findings to vary by sex.

### Study participants.

This study included previously collected paired core liver biopsies from 10 men with HIV and CHB (20 total biopsies) enrolled in the HIV-HBV Cohort Ancillary Study of the Hepatitis B Research Network at Johns Hopkins University (31). The paired liver biopsies were obtained a median of 3.7 years apart. At the time of biopsy 1, 4 individuals had HBeAg-negative CHB and 6 individuals had HBeAg-positive CHB, including 1 individual who underwent HBeAg seroconversion about 6 months after biopsy 1 (HB11). Participants were on varying durations of nucleos(t)ide analog (NUC) treatment (see Results; Table 1) as part of their antiretroviral therapy, which included tenofovir disoproxil fumarate, emtricitabine, or entecavir. We classified people by their exposure to NUCs prior to biopsy 1: an early group and a prolonged group (defined in Table 1). HBV DNA levels between biopsies 1 and 2 are shown in Supplemental Figure 3.

### Single-cell laser capture microdissection and DNA/RNA extraction.

At the time of biopsy, liver tissues were immediately placed into neutral optimal cutting temperature and stored in liquid nitrogen until use. Tissues were cryosectioned at 10 μm thickness onto polyethylene naphthalate (PEN) membrane slides; single-hepatocyte equivalents were individually isolated in a grid fashion, as previously described (5–7). Each hepatocyte was deposited into a microcentrifuge tube with proprietary lysis buffer (ZR-Duet DNA/RNA MiniPrep kit, Zymo Research). RNA and DNA were separately extracted, including a DNase I in-column digestion to purify RNA, as previously described (5–7). Total complementary DNA (cDNA) was synthesized from RNA using oligo-dT and random hexamer priming with the Superscript IV First-Strand Synthesis System (Thermo Fisher Scientific). An abundant host cytoplasmic RNA, 7SL, was measured in every capture using real-time reverse-transcription quantitative PCR to assess for cell fragmentation. Captures within 1 standard deviation below a negative control cycle threshold were excluded from the final analysis, as previously described (5–7).

After the quality control assessment to filter out cell fragments, a median of 172 single-cell equivalents were analyzed per biopsy with 3,436 hepatocytes analyzed in 20 biopsies. For this study, a cell was considered transcriptionally active if there were detectable ddPCR targets (Figure 1).

### Multiplex ddPCR for cccDNA-derived and iDNA-derived S transcripts.

Our multiplex ddPCR assay targets 2 amplicons along the HBV transcriptome (Figure 1A); we exploited the abundance of viral-human chimeric junctions in mRNA that derives from iDNA that frequently lack the canonical polyadenylation signal (PAS). The mid-HBV amplicon (nt 253–418) captures transcripts derived from both cccDNA and iDNA (specifically the 3.5 kb, 2.4 kb, and 2.1 kb transcripts), whereas the 3′-HBV amplicon (nt 1774–1881) is just upstream of the PAS (around 40 nt) and largely captures cccDNA-derived transcripts rather than iDNA-derived transcripts (Figure 1), as previously described (2). cDNA along with primers/probes targeting the mid-HBV and 3′-HBV ddPCRs was performed using the following cycling parameters: 1 cycle of 94°C for 10 minutes, 40 cycles of 94°C for 30 seconds and 57°C for 1 minute, 1 cycle of 98°C for 10 minutes, 1 cycle of 12°C for 10 minutes, and 1 continuous cycle at 4°C until reading, as previously described (5–7). Plates were read on the QX200 Droplet Reader (Bio-Rad), which provides results of the copies of each amplicon per microliter of reaction.

### Analysis.

We defined cells as transcriptionally active if they had positive droplets for either the mid-HBV or 3′-HBV assays. For each transcriptionally active cell, we calculated an iDNA transcriptional index (iDNA-TI) using the ratio of the number of copies of the mid-HBV to 3′-HBV amplicons, as previously described (2). Since the 3′-HBV amplicon only detects cccDNA-derived transcripts, we classified viral transcription in each cell as chiefly cccDNA-derived (iDNA-TI ≤ 1), mixed cccDNA- and iDNA-derived (iDNA-TI > 1), or chiefly iDNA-derived (only mid-HBV^+^). Chimericseq was applied to RNA-Seq data from the same people to identify virus-host junctions (Supplemental Figure 1) (36).

### Immunohistochemistry.

Glass slides were stained at the Johns Hopkins Pathology Center with immunohistochemical stains for hepatitis B core (HBcAg) and surface antigens, and these were processed and stained as previously described (6, 7).

### Serological assessment.

Frequent plasma sampling was performed in tandem with biopsies and tested for qHBsAg using the Roche Diagnostics Elecsys platform according to the manufacturer’s instructions. HBV viral load was determined at the Johns Hopkins Clinical Laboratory using the Roche COBAS TaqMan assay. Units for both were reported as log_10_ international units per milliliter (IU/mL).

### Statistics.

Treatment groups were compared with one another using the Wilcoxon’s rank-sum test. To compare the levels of different transcripts per cell (mid-HBV or 3′-HBV) across people and time (biopsy 1 and 2), we used linear mixed-effects models (with participant as the random factor) using the R package lmerTest v3.1.3 (32), because of the hierarchical repeated nature of the data (measurements for multiple cells within individuals). Comparing levels of transcripts within people between biopsies 1 and 2, we used a pairwise Wilcoxon’s rank-sum test in R (“pairwise.wilcox.test”). Spearman’s correlation coefficient analyses were performed to assess relationships between variables. Fisher’s unconditional exact (uncondExact) 2 × 2 function was used to analyze changes in proportions of cells within individuals. All statistical tests were done using R Statistical Software v4.2.2 (33). A *P* value less than 0.05 was considered statistically significant.

We calculated decline rates of the fraction of cells infected with predominance of different transcripts using nonlinear mixed-effects models with the software Monolix (Lixoft) (34). We tested 3 different models to describe the decay data across all individuals (early and prolonged treatment). In the first model, we assumed that the decay of the fraction of cells followed a single exponential decay across all individuals (including those in the early and prolonged groups), which implies a single population of infected cells decaying over the time span of the analyses. The second model assumed that the decay was biexponential across all, which can be interpreted as 2 populations of cells decaying at different rates from the start of treatment. The first population is dominant at early times and decays faster, whereas the second, slower, cell population dominates at later times. The third model assumed that the early and prolonged treatment groups had separate independent single exponential decays, which could indicate that there are 2 different (at least in terms of decay rates) populations of cells early and later in treatment. The models were compared with the corrected Bayesian Information Criteria (cBIC), as provided by Monolix, where lower cBIC corresponds to a better fit (35). Note that we calculated the fraction of infected cells (with mid-HBV or 3′-amplicons) over all cells. Since we can assume that the total number of hepatocytes is stable within each individual (the liver size is tightly controlled), the decay rate of the fraction of cells is equivalent to the decay rate of total cells containing those mRNA species. On the other hand, proportions of cells in different transcriptional classes (e.g., chiefly cccDNA) were calculated over infected cells, i.e., transcriptionally active cells. For completeness, we also tested a simpler linear decay model, which did not accurately describe the data. The best model (lower cBIC) for almost all cases was the one assuming 2 independent single exponential decays for the early and prolonged groups, which is what we present in Results.

### Study approval.

This study was reviewed and approved by the Office of Human Subjects Research Institutional Review Board, IRB-3, Baltimore, Maryland, USA. Participants gave written informed consent for use of their tissues for research purposes through the Hepatitis B Research Network.

### Data availability.

Data can be accessed through Vivli (www.vivli.org). Values for all data points in the figures can be found in the Supporting Data Values file.

## Author contributions

MT, DLT, CLT, and AB conceptualized the study. MT, TG, GB, MA, HSH, MM, CML, YZ, RMR, CLT, and AB developed the methodology. MT, TG, GB, MA, MM, CML, NE, YZ, RMR, CLT, and AB carried out the investigation. MT, GB, MA, RMR, CLT, and AB conducted analyses. MT, GB, CLT, and AB contributed to visualization and generated figures. GC, MSS, RKS, RMR, CLT, and AB acquired funding. MT, TG, GB, MA, GC, HSH, MM, CML, NE, MSS, RKS, YZ, RMR, DLT, CLT, and AB wrote the manuscript draft or reviewed and edited it.

## Supplementary Material

Supplemental data

Supporting data values

## Figures and Tables

**Table 1:**
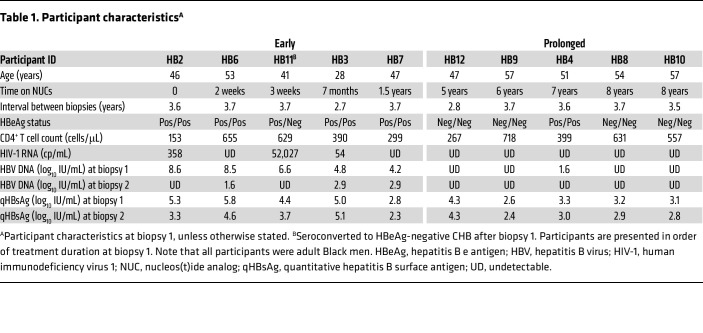
Participant characteristics^A^

## Version 1 02/03/2025

In-Press Preview

## Version 2 03/17/2025

Electronic publication
