# Supplementary material for: Transcription of hepatitis B surface antigen shifts from cccDNA to integrated HBV DNA during treatment
Source: J Clin Invest. 2025 Jan 30;135(6):e184243. doi: 10.1172/JCI184243 (PMC11910228; doi:10.1172/JCI184243)
Supplement: Supplemental data [file jci-135-184243-s036.pdf]

SUPPLEMENTARY MATERIAL

Supplementary Methods

Chimera Analysis

To confirm that our 3’ HBV amplicon would exclude iDNA-derived transcripts, we identified viral junctions by performing RNA sequencing on permeabilized liver tissue through the 10x Visium Spatial Transcriptomics technology pipeline from 9/10 participants at biopsy 1. Using the ChimericSeq Software™, HBV-human chimeric reads that are indicative of mRNA transcribed from integrated HBV DNA were moved forward for analysis: all non-chimeric human and HBV reads with a homology percentage threshold of 95% were filtered out for the analysis (36). After excluding duplicate reads, reads with poor quality scores, and normalizing the data for a threshold of >1 read per nucleotide position, we counted all the reads that had unique chimeric junctions at each nucleotide position along the HBV genome (Supp Fig. 1).

Isoform Analysis

Prototype Research Use Only (RUO) chemiluminescent microparticle immunoassays (CMIA) were tested on the fully automated ARCHITECT i2000sr platform for the detection of HBsAg isoforms were conducted for each timepoint on each of the 10 study participants. Briefly, the three HBsAg isoforms assays use mouse monoclonal antibodies specific for each of PreS1, PreS2, and S to detect Large-, Middle-, and Total-HBsAg (which detects all three isoforms), respectively. The isoform assays report results as a signal/noise ratio (S/N) (17, 18).

Supplementary Table

Table of p-values for changes in the numbers of cells chiefly transcribing from different templates using individual data and Fisher exact test.

	HB6	HB7	HB3	HB2	HB11
cccDNA	$5x10^{-5}$	$<10^{-5}$	0.002	0.47	$<10^{-5}$
iDNA	$<10^{-5}$	$<10^{-5}$	0.044	0.0062	0.015
Mixed	$<10^{-5}$	0.176	0.017	0.0002	$<10^{-5}$
	HB4	HB8	HB9	HB10	HB12
cccDNA	0.083	0.005	>0.99	>0.99	>0.99
iDNA	0.29	0.002	>0.99	>0.99	>0.99
Mixed	0.26	0.30	>0.99	>0.99	>0.99

Supplementary Figures

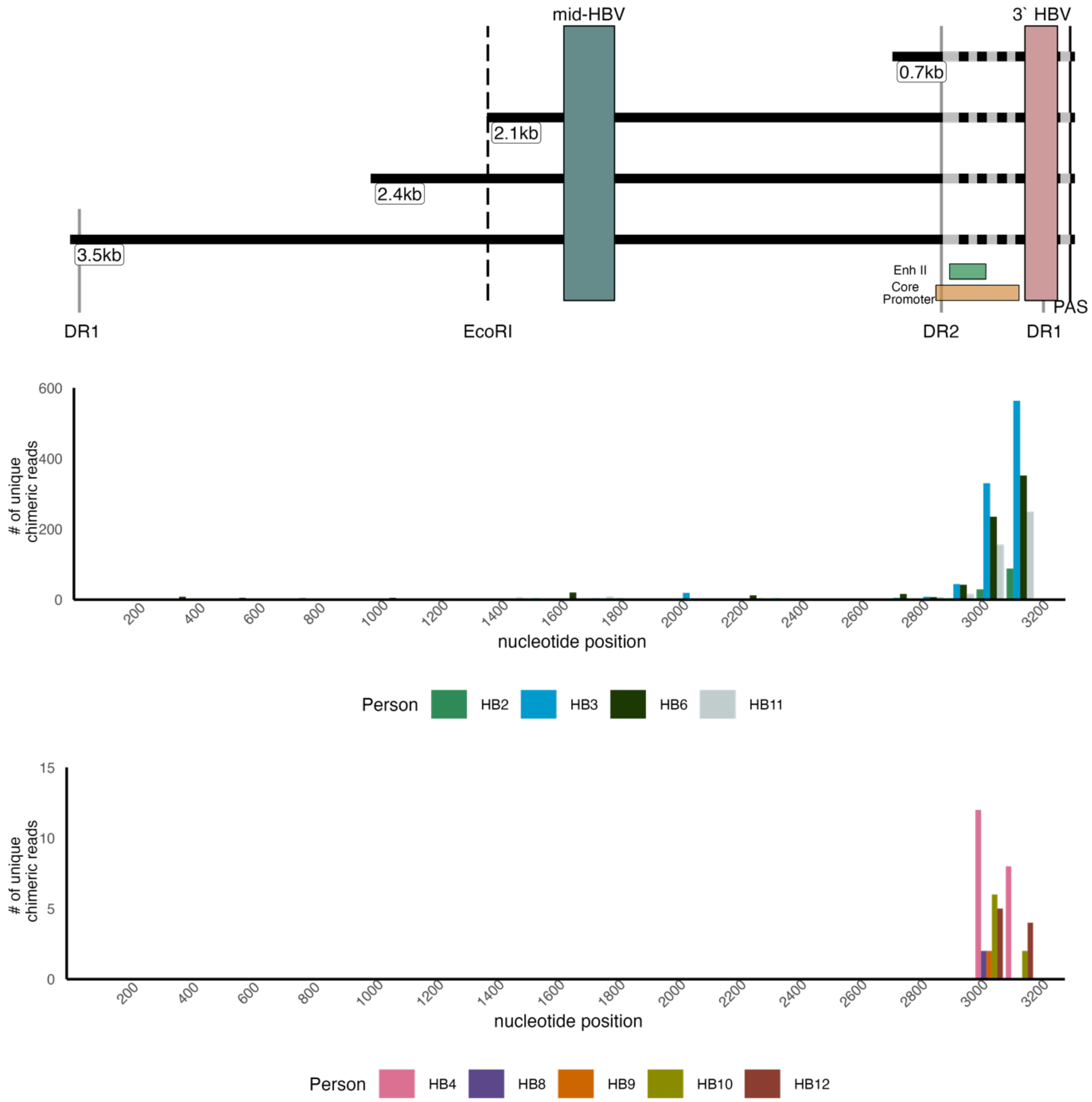

Figure S1. iDNA-derived viral-human chimeric mRNA junctions. (A) Horizontal lines depict the four canonical HBV mRNAs produced by each of the open reading frames. The variable chimeric virus-host regions are displayed as hashed lines at the 3' end of transcripts. Solid vertical lines show the positions of the DR2, DR1, and the canonical poly A signal (PAS),

respectively. The colored boxes depict the two ddPCR target amplicons: mid-HBV and 3'HBV. The locations of the HBV Enhancer II (EnhII) and core promoter regions are displayed as green and orange boxes, respectively. Dotted line represents EcoRI cut site which is included for reference. **(B)** HBV-human chimeric junction analysis was performed on 9/10 participants at biopsy 1. After excluding duplicate reads and normalizing the data for a threshold of >1 read, we counted all unique chimeric reads at each nucleotide position along the HBV genome, indicating the location in the genome map of the HBV-human junction: top panel represents 4/5 early treatment biopsies and the bottom panel represents all 5 prolonged treatment biopsies. Note differences in y-axis scale for the prolonged treatment biopsies due to fewer total HBV reads compared to the early treatment biopsies.

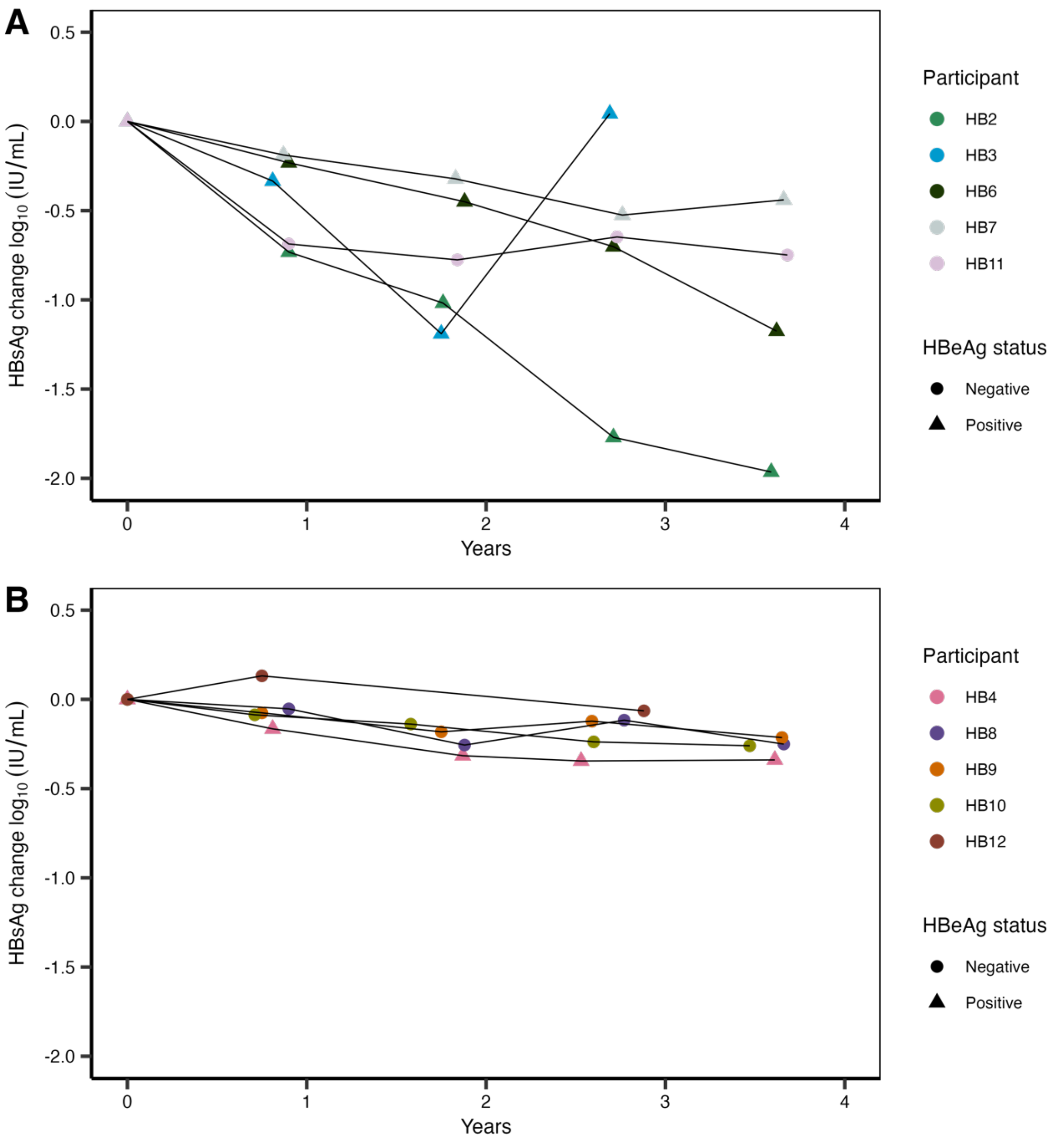

Figure S2. Quantitative HBsAg (qHBsAg) trajectories in individual participants. Blood qHBsAg *changes* relative to biopsy 1 are shown; each person is represented by a separate line. (**A**) Participants in *early* treatment group exhibited >0.5 $\log_{10}$ IU/mL decline in HBsAg during the study period. **(B**) Participants in *prolonged* treatment group did not experience declines in qHBsAg. The limit of detection (LOD) for the qHBsAg testing was 0.05 IU/mL. Different

shapes are used to represent HBeAg status at the time of sampling. Note: one participant, HB3, experienced an interruption in treatment between biopsies.

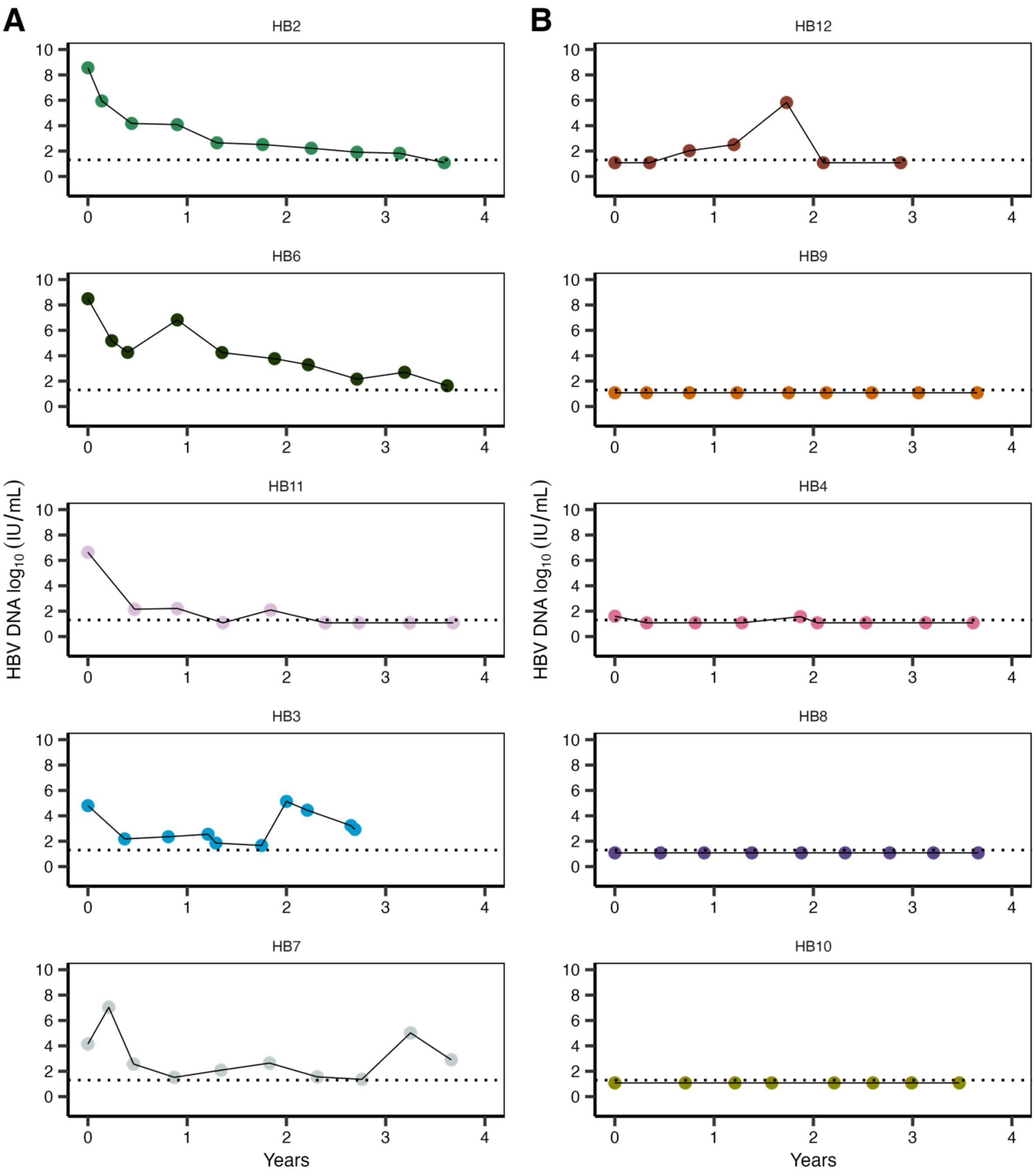

Figure S3. Plasma HBV DNA levels in each participant. $\log_{10}$ IU/mL HBV DNA levels in blood over the course of the study are shown. The first and final data points of each line represent biopsies 1 and 2, respectively. Participants are presented in order of NUC duration at the time of biopsy 1 in (**A**) *early* treatment group and **(B)** *prolonged* treatment. The limit of

detection (LOD) for the HBV DNA testing here is represented by the dotted line at 1.3 $\log_{10}$ IU/mL (20 IU/mL).

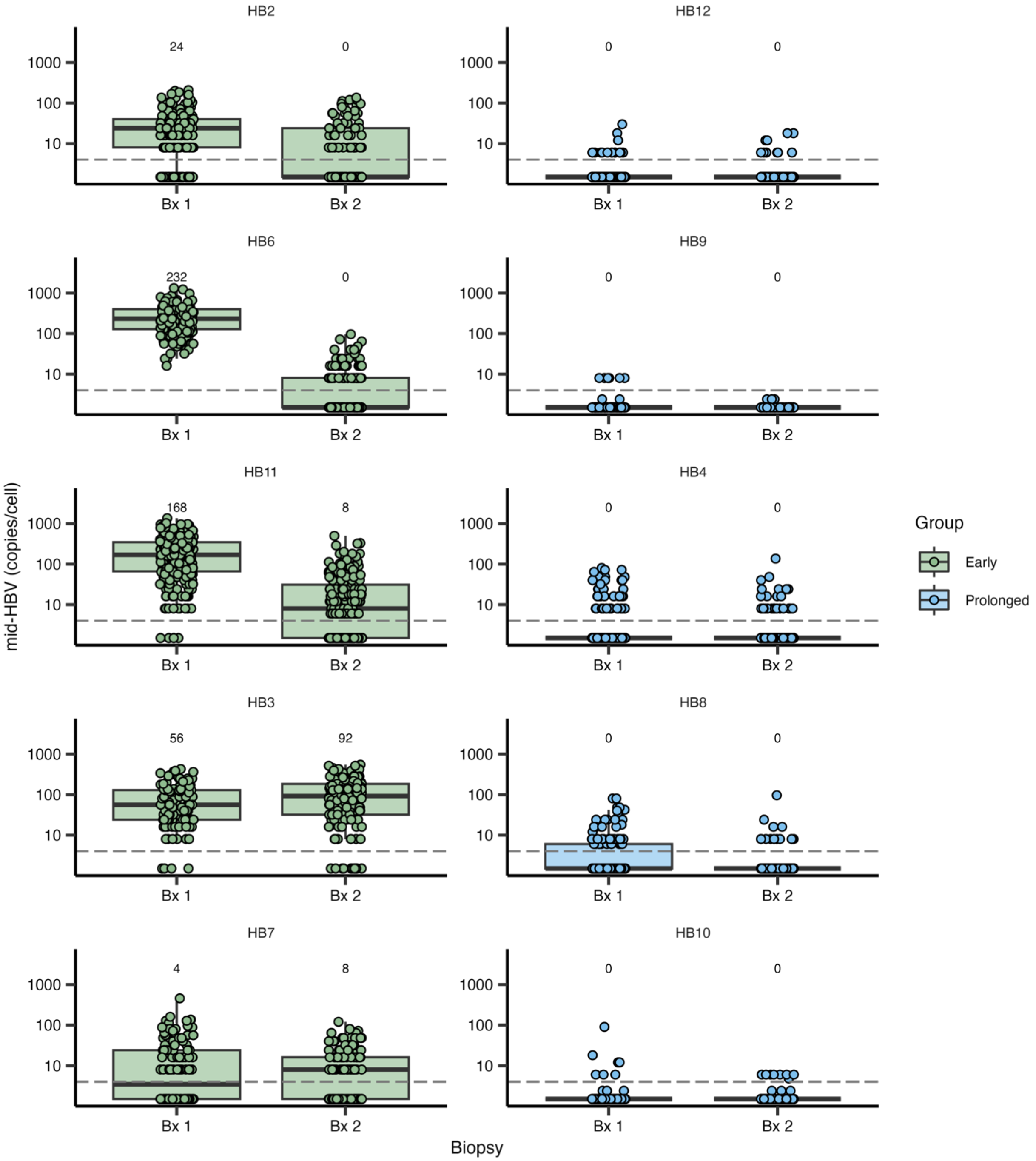

Figure S4. Mid-HBV quantities at each biopsy in each participant. Participants are presented in order of NUC duration: median copies/cell are displayed above the box plot. Limit of detection is represented by the dashed line while cells with no detectable mid-HBV were arbitrarily assigned a value of 1.5 copy/cell for visualization on a $\log_{10}$ scale. The p-values

comparing biopsy 1 and 2 quantities are as follows: HB2 ($P<.0001$), HB6 ($P<.0001$), HB11 ($P<.0001$), HB3 ($P=.006$), HB7 ($P=0.65$), HB12 ($P=0.21$), HB9 ($P=0.67$), HB4 ($P=0.49$), HB8 ($P<.0001$), HB10 ($P=0.97$). Abbreviations: Bx, biopsy.

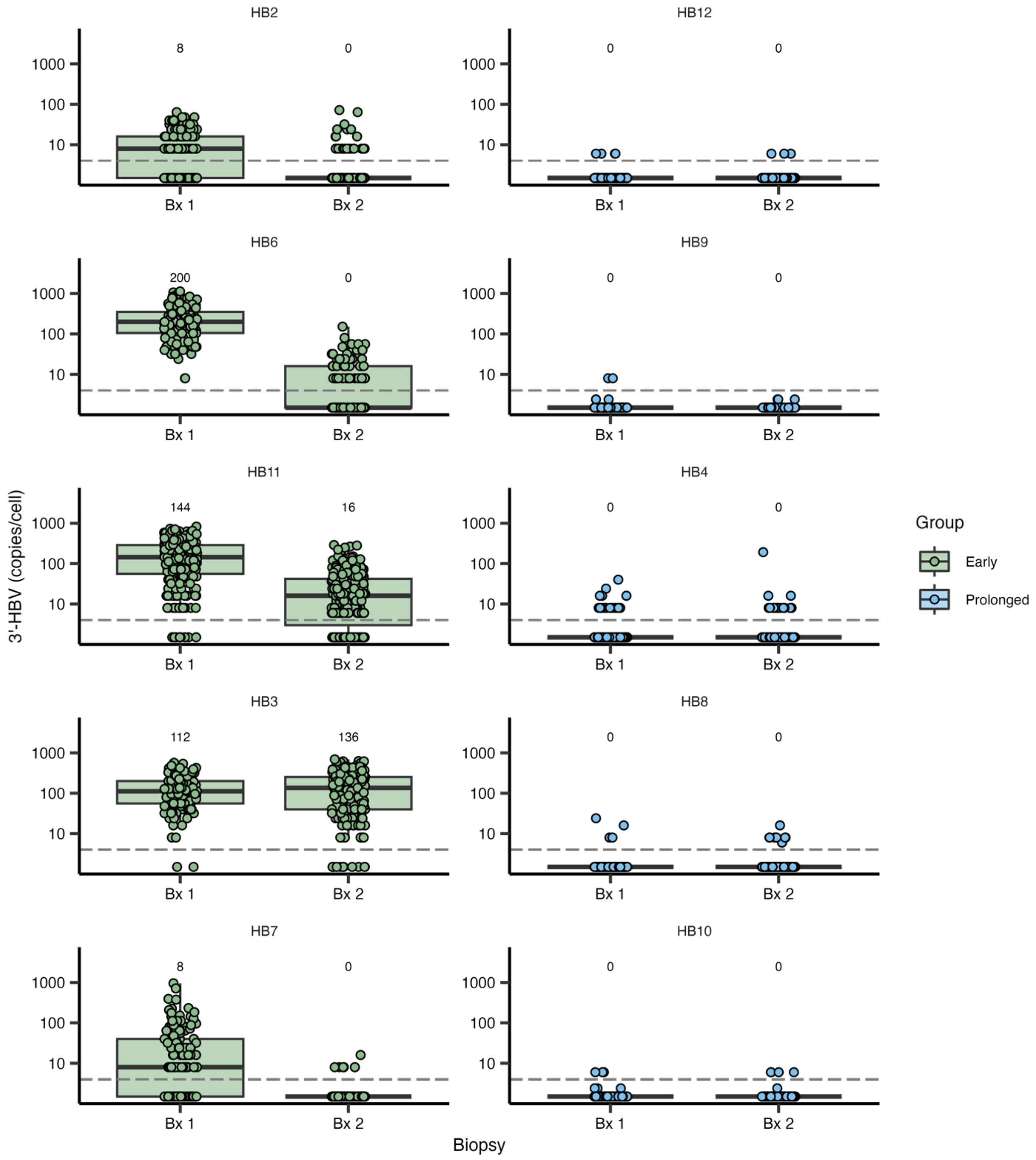

Figure S5. 3'-HBV quantities at each biopsy in each participant. Participants are presented in order of NUC duration; median copies/cell are displayed above the box plot. Limit of detection is represented by the dashed line while cells with no detectable 3'-HBV were arbitrarily assigned a value of 1.5 copy/cell for visualization on a $\log_{10}$ scale. The p-values comparing biopsy 1 and 2

quantities are as follows: HB2 ($P<.0001$), HB6 ($P<.0001$), HB11 ($P<.0001$), HB3 ($P=0.34$), HB7 ($P<.001$), HB12 ($P=0.75$), HB9 ($P=0.58$), HB4 ($P=0.27$), HB8 ($P=0.17$), HB10 ($P=0.65$).

Abbreviations: Bx, biopsy.

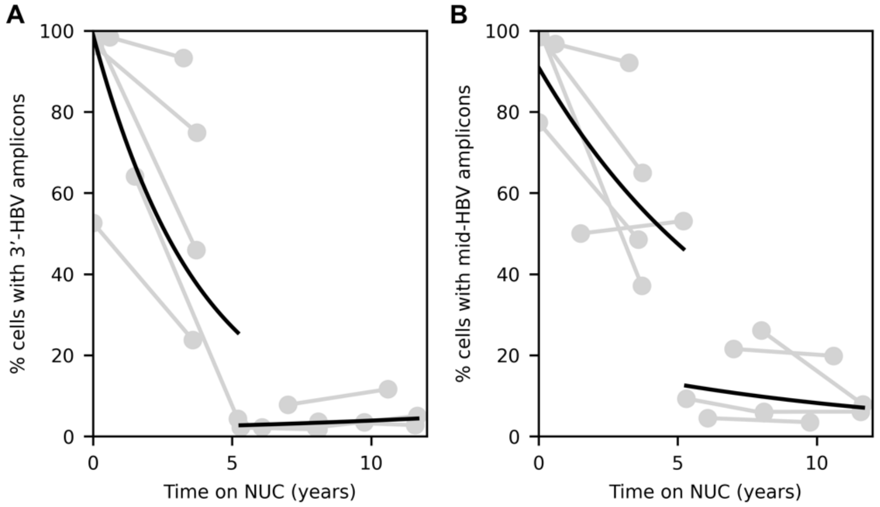

Figure S6. Decay rates of cell populations with each ddPCR amplicon. Each grey line represents a participant with points corresponding to biopsies 1 and 2. The time on NUCs (years) is plotted against the proportion of *total* cells with detectable **(A**) Mid-HBV and **(B**) 3'-HBV. Mixed-effects modeling was used to generate rates across the group, represented by the black lines (see Methods).

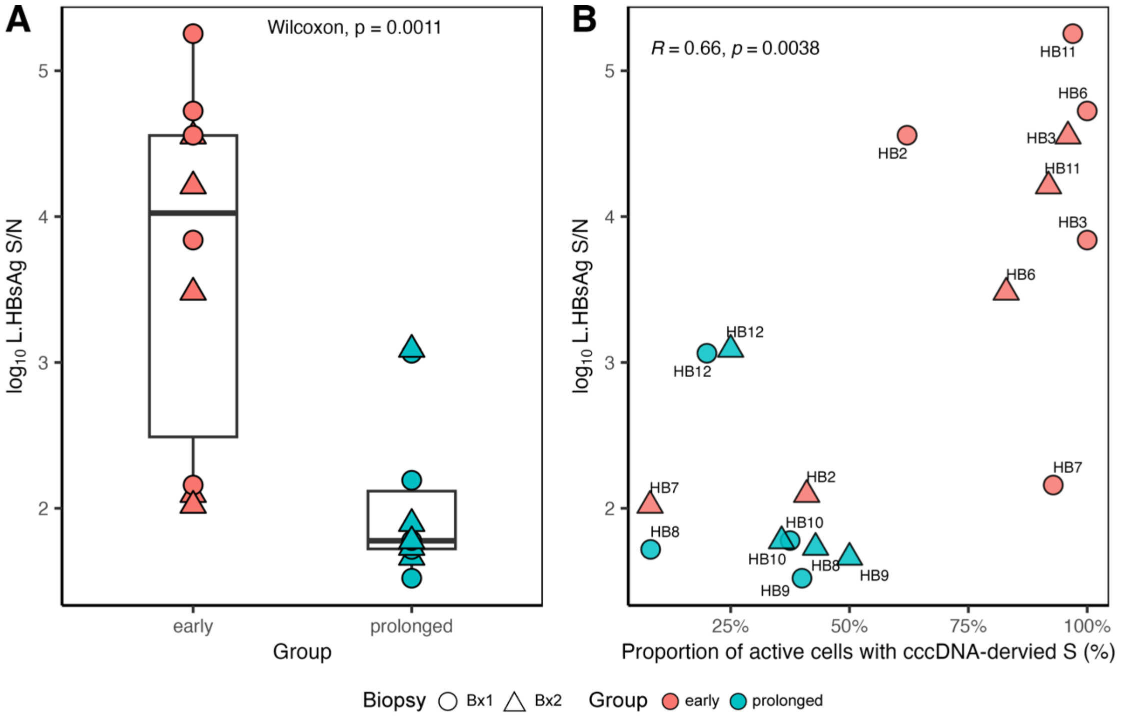

Figure S7. The proportion of intrahepatic cccDNA-derived HBV transcripts is associated with L-HBsAg in blood. (**A**) Abundance of L-HBsAg was compared in biopsies from people in *early* treatment group (red) vs. *prolonged* group (blue). Wilcoxon rank-sum test was used to compare the quantities of L-HBsAg between groups. The amount of L-HBsAg was measured by the signal/noise (S/N) ratio as determined by binding to the PreS1 antibody. (**B**) The percentage of *transcriptionally-active* cells with any cccDNA-derived HBV transcripts (including those with chiefly cccDNA derived transcription and those with mixed) was correlated with $\log_{10}$(L-HBsAg S/N). Spearman's correlation coefficient and associated P value is shown. HB4 was excluded due to inconsistencies in signal recovery for the isoform analysis. Abbreviations: Bx, biopsy.

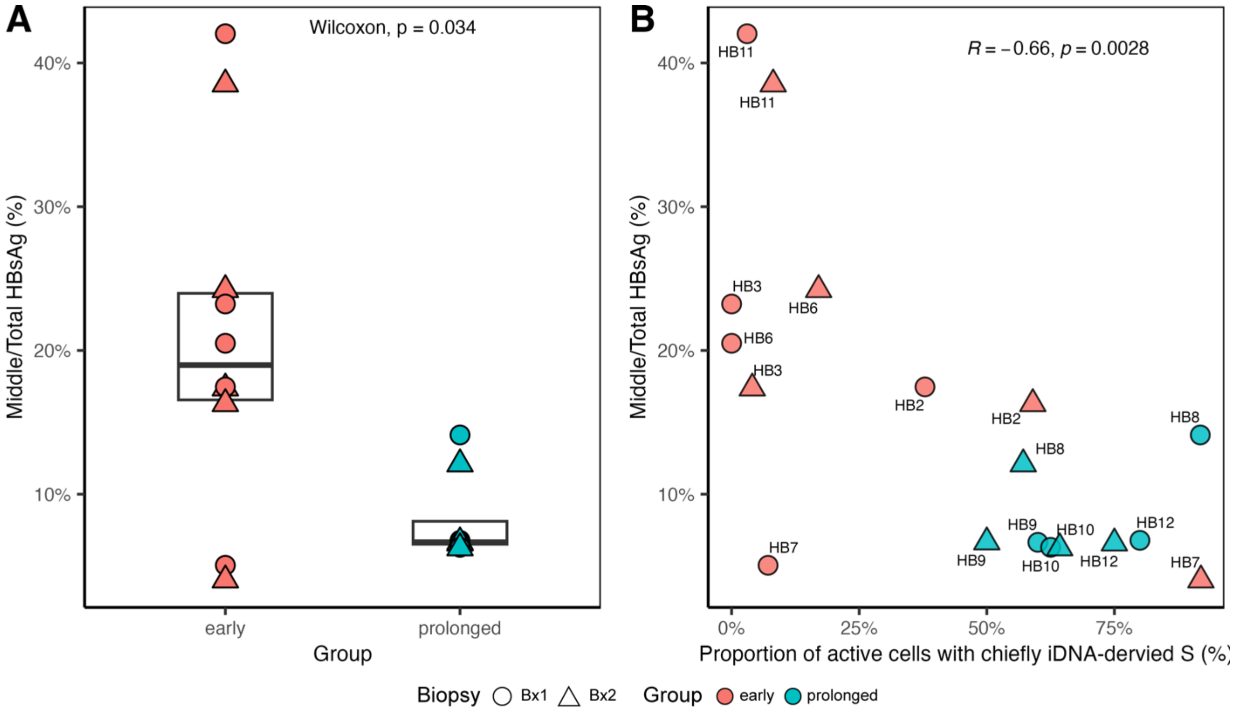

Figure S8. Proportion of cells with chiefly iDNA-derived transcripts correlated with Sm-HBsAg. (A) The proportion of total HBsAg that was Middle (M)-HBsAg as an estimate of (Sm)all HBsAg (M/T) was calculated for each participant and compared in biopsies from people in early treatment group vs. prolonged group. Wilcoxon rank-sum test was used to compare between groups. (**B**) The percentage of all single cells with chiefly iDNA-derived HBV transcripts was correlated with the proportion of M/T (as a measure of Sm-HBsAg) in blood at that biopsy. Spearman's correlation coefficient and associated P value is shown. HB4 was excluded due to inconsistencies in signal recovery for the isoform analysis. Abbreviations: Bx, biopsy. M/T, the proportion of Total HBsAg that is attributable to M-HBsAg.
